# Comparison of diagnostic accuracy for eight SARS-CoV-2 serological assays

**DOI:** 10.11613/BM.2021.010708

**Published:** 2021-02-15

**Authors:** Andrea Tešija Kuna, Milena Hanžek, Ines Vukasović, Nora Nikolac Gabaj, Valentina Vidranski, Ivana Ćelap, Marijana Miler, Nevenka Stančin, Brankica Šimac, Marcela Živković, Marko Žarak, Marta Kmet, Marijana Jovanović, Sanja Tadinac, Sandra Šupraha Goreta, Josipa Periša, Ivan Šamija, Mario Štefanović

**Affiliations:** 1 Department of Clinical Chemistry, Sestre milosrdnice University Hospital Center, Zagreb, Croatia; 2 Department of Oncology and Nuclear Medicine, Sestre milosrdnice University Hospital Center, Zagreb, Croatia; 3 Clinical Department of Laboratory Diagnostics, University Hospital Dubrava, Zagreb, Croatia; 4 Department of Biochemistry and Molecular Biology, Faculty of Pharmacy and Biochemistry, University of Zagreb, Zagreb, Croatia

**Keywords:** sensitivity, specificity, SARS-CoV-2, serological test

## Abstract

**Introduction:**

Severe acute respiratory syndrome coronavirus 2 (SARS-CoV-2) serological tests have been suggested as an additional diagnostic tool in highly suspected cases with a negative molecular test and determination of seroprevalence in population. We compared the diagnostic performance of eight commercial serological assays for IgA, IgM, and IgG antibodies to the SARS-CoV-2 virus.

**Materials and methods:**

The comparison study was performed on a total of 76 serum samples: 30 SARS-CoV-2 polymerase chain reaction (PCR)-negative and 46 SARS-CoV-2 PCR-positive patients with asymptomatic to severe disease and symptoms duration from 3-30 days. The study included: three rapid lateral flow immunochromatographic assays (LFIC), two enzyme-linked immunosorbent assays (ELISA), and three chemiluminescence immunoassays (CLIA).

**Results:**

Agreement between IgM assays were minimal to moderate (kappa 0.26 to 0.63) and for IgG moderate to excellent (kappa 0.72 to 0.92). Sensitivities improved with > 10 days of symptoms and were: 30% to 89% for IgM; 89% to 100% for IgG; 96% for IgA; 100% for IgA/IgM combination; 96% for total antibodies. Overall specificities were: 90% to 100% for IgM; 85% to 100% for IgG; 90% for IgA; 70% for IgA/IgM combination; 100% for total antibodies. Diagnostic accuracy for IgG ELISA and CIA assays were excellent (AUC ≥ 0.90), without significant difference. IgA showed significantly better diagnostic accuracy than IgM (P < 0.001).

**Conclusion:**

There is high variability between IgM assays independently of the assay format, while IgG assays showed moderate to perfect agreement. The appropriate time for testing is crucial for the proper immunity investigation.

## Introduction

A newly identified virus belonging to the β-subgroup of the *Coronaviridae* family caused the 2019 global pandemic of the infectious disease marked with severe acute respiratory distress syndrome (*1*). The new virus is 96% identical at the whole-genome level to a bat coronavirus and shares 80% identity at a nucleotide level with another highly pathogenic coronavirus responsible for acute respiratory syndrome outbreak in 2003, severe acute respiratory syndrome coronavirus (SARS-CoV) (*2*). Consequently, the new coronavirus was named SARS-CoV-2 by the International Committee of Taxonomy of Viruses (ICTV) and the disease, Coronavirus disease 2019 (COVID-19) (*1*, *3*).

Coronaviruses are positive-sense single-stranded RNA viruses with spherical encapsulated particle structure. The viral envelope (E) is a lipid bilayer where membrane glycoproteins (M) and spike proteins (S) are anchored. The interior of the particle contains nucleocapsid (N) proteins bound to the RNA molecule (*4*). Spike glycoprotein is a transmembrane protein responsible for the crown-like appearance (corona) of the virus particle and contains two functional subunits: S1 and S2. The S1 subunit is responsible for binding to the host-cell receptor, angiotensin-converting enzyme 2 (ACE2) *via* receptor-binding domain (RBD), while the S2 subunit contains elements essential for fusion of the virus (*1*, *4*). The incubation period usually lasts from 4–6 days, with the onset of symptoms within 14 days in 95% of cases (*1*). Clinical presentation varies from asymptomatic through mild and moderate symptoms which include cough, fever, shortness of breath, asthenia, arthralgia, myalgia, anosmia, and ageusia to the very severe and critical cases of severe pneumonia, septic shock, and acute respiratory distress syndrome (ARDS) (*1*, *5*).

Currently, the gold standard for diagnosis of SARS-CoV-2 infection is the detection of the viral sequence by reverse transcriptase polymerase chain reaction (RT-PCR) on respiratory tract specimens (usually from the upper respiratory tract taken by oropharyngeal or nasopharyngeal swabs). Unfortunately, the sensitivity of this method is highly dependent on preanalytical and analytical variables such as the type of the specimen (upper or lower respiratory tract), sampling technique, the timing of the sampling, or conditions during transportation of the sample, and false-negative results can be attributed to all of these (*1*, *6*). According to current opinion, diagnostic use of serological tests has limited use as an additional aid to molecular testing for patients who are highly suspected for infection but repeatedly negative on molecular testing and in deciding on the discharge of patients recovered from COVID-19 but still RT-PCR positive (*7*). In an immunocompetent individual, the production of virus-specific host antibodies of IgA and IgM isotype is consistent with an acute phase infection, while IgG isotype appears with the later phase of infection. The diagnostic relevance of the serological test is highly dependent on the proper timing of sampling. However, the kinetics of immunologic response in SARS-CoV-2 infection is still unclear, as well as the duration of persistence and the protective role of circulating antibodies (*8*). Highly variable median seroconversion times across studies have been reported; for IgM 5–17 days and for IgG 6–14 days, which can be attributed to different study populations, the timing of sample, but also to different performances of the used assays (*9*).

In response to an urgency, a huge number of commercial serological tests in different formats have been rapidly developed with unknown or questionable clinical performance. This primarily refers to the numerous rapid easy-to-use devices offering combined detection of IgM and IgG antibodies to facilitate use outside of limited laboratory capacities. Unfortunately, many of these tests are not properly validated to accomplish the proposed role. There is an emerging number of articles with topics on the diagnostic performance of various SARS-CoV-2 serological assays including rapid test devices and newly developed assays of standard format like enzyme-linked immunosorbent assays (ELISA) and chemiluminescence immunoassays (CLIA) (*10*-*14*).

Our study aimed to compare the diagnostic performance of eight different commercial serological assays for the detection of IgA, IgM, and IgG antibodies to the SARS CoV-2 virus with three assays compared for the first time to other assays included in the study.

## Materials and methods

### Subjects

The diagnostic accuracy and comparison study were performed in June 2020 in the Department of Clinical Chemistry and the Laboratory of Department of Oncology and Nuclear Medicine Sestre Milosrdnice University Hospital Centre, Zagreb. The collection of samples was performed during April and May 2020 in Sestre Milosrdnice University Hospital Centre and Clinical Department of Laboratory Diagnostics, University Hospital Dubrava, Zagreb. The inclusion criteria were the availability of the SARS-CoV-2 RT-PCR result and anamnestic data. This study was done using leftover serum samples from routine chemistry testing, taken either the same day when the patients were referred to the RT-PCR SARS-CoV-2 testing or during the hospitalization due to the COVID-19 disease. A total of 76 samples were included: 30 samples from SARS-CoV-2 RT-PCR-negative patients and 46 SARS-CoV-2 RT-PCR-positive patients (32 in case of Lumiquick rapid test IgM/IgG). The sera were stored at - 80 °C until analysis. Repetitive thawing was avoided. The clinical presentation among the patients in the positive group was different (from asymptomatic to very severe) as well as the duration of the symptoms (from 3 to 30 days). Eleven persons (11/46) were asymptomatic, 27 out of 46 had symptoms lasting longer than 10 days and 8 out of 46 had symptoms lasting shorter than 10 days. For one patient, data on the duration of symptoms were not completely clear in the sense that could be classified with certainty as less than or more than 10 days. For 10 out of 11 asymptomatic persons, the blood sampling was performed one day after the RT-PCR confirmed infection. SARS-CoV-2 PCR-negative patients (N = 30) included the patients referred for RT-PCR SARS-CoV-2 testing before hospitalization in Sestre Milosrdnice University Hospital Centre due to different indications and did not have symptoms associated with COVID-19 nor evidence of the previous SARS-CoV-2 infection.

The Ethical Board of the Sestre Milosrdnice University Hospital Centre approved the study.

### Methods

#### Serological assays

Eight different serological tests for IgA, IgM, or IgG antibodies to SARS CoV-2 virus detection were compared. Three of them were rapid lateral flow immunochromatographic assays (LFIC): Biozek COVID-19 IgG/IgM Rapid cassette (Biozek medical, Inzek B.V., Apeldoorn, The Netherlands), Encode COVID-19 IgG/IgM Rapid test device (Zhuhai Encode Medical Engineering Co., Zhuhai, China), and Lumiquick rapid test IgM/IgG (LumiQuick Diagnostics, Santa Clara, USA). Two of them were ELISA tests: Euroimmun IgA/IgG Anti-SARS-CoV-2 (Euroimmun, Lübeck, Germany), and ELISA Vircell IgM+IgA/IgG (Vircell, S. L. Parque Tecnologico de la Salud, Spain). Three fully automated assays were: the CLIA MAGLUMI 2019-nCoV IgG/IgM on Snibe Maglumi 800 analyser (both Snibe, Shenzhen New Industries Biomedical Engineering, Shenzhen, China), chemiluminescent microparticle immunoassay (CMIA) Abbott SARS-CoV-2 IgG on Abbott Architect i2000SR analyser (both Abbott Laboratories, Abbott Park, Chicago, USA) and electrochemiluminescence immunoassay (ECLIA) Roche Elecsys Anti-SARS-CoV-2 total antibody assay on Cobas e601 analyser (both Roche Diagnostics, Santa Clara, USA). All assays have an *in-vitro* diagnostics certificate (CE-IVD). Detailed assay specifications are given in Table 1. All assays were performed according to the manufacturer’s instructions. For each method and every run, the quality control recommended by the manufacturer was performed. The results of all quality control were within the recommended range.

**Table 1:** Detailed assay specifications

**Assay**	**Biozek COVID-19 IgG/IgM Rapid cassette**		**ENCODE COVID-19 IgG/IgM Rapid test device**		**Lumiquick rapid test**		**Euroimmun IgA/IgG_Anti-SARS-CoV-2**				**Vircell IgM+IgA/IgG**		**MAGLUMI 2019-nCoV IgG/IgM**					**Abbott SARS-CoV-2 IgG**	**Elecsys Anti-SARS-CoV-2**
Parameter	IgM	IgG	IgM	IgG	IgM	IgG	IgA	IgG			IgA+IgM	IgG	IgM			IgG		IgG	Total antibodies
Method	LFIC		LFIC		LFIC		ELISA				ELISA		CLIA					CMIA	ECLIA
Reagent stability after opening	NA		NA		until expiration date		until expiration date				until expiration date		6 weeks					until expiration date	72 hours
Test number *per* one pack	30		25		25		96				96		100					100 or 500	200
Specimens	whole blood, serum, plasma		whole blood, serum, plasma		whole blood, serum, plasma		serum, plasma				serum, plasma		serum, plasma					serum, plasma	serum, plasma
Sample volume *per* test (µL)	10 (for both antibodies)		10 (for both antibodies)		2		10				5		10					75	20
Sample dilution	NA		NA		NA		1: 101				1: 21	1: 100	1: 9					NA	NA
Test duration (min)	10 (for both antibodies)		10 (for both antibodies)		15 (for both antibodies)		120				120		35					29	20
Result type, units	qualitative		qualitative		qualitative		semiquantitative, ratio				semiquantitative, antibody index (AI)		qualitative, AU/mL					qualitative, ratio	qualitative, cut-off index (COI)
Negative result	control line only		control line only		control line only		0.8				< 6	< 4	< 1.0					< 1.4	< 1.0
Borderline result	NA		NA		NA		≥ 0.8 to < 1.1				6-8	4-6	NA					NA	NA
Positive result	control line + IgM line	control line + IgG line	control line + IgM line	control line + IgG line	control line + IgM line	control line + IgG line	≥ 1.1				> 8	> 6	≥ 1.0					≥ 1.4	≥ 1.0
Antigen	not-specified		not-specified		not-specified		S1 domain of the spike protein of SARS-CoV-2 expressed recombinantly in the human cell line HEK 293				SARS CoV-2 inactivated antigen		2019-nCoV recombinant antigen					nucleocapsid protein	recombinant nucleocapsid Ag
Control material	control line to confirm that the test is functional and indicates the proper liquid flow through the strip		control line to confirm that the test is functional and indicates the proper liquid flow through the strip		control line to confirm that the test is functional and indicates the proper liquid flow through the strip		positive and negative human IgA controls (provided within the kit)			positive and negative human IgG controls (provided within the kit)	positive and negative human IgM+IgA controls, IgM+IgA cut-off control	positive and negative human IgG, IgG cut-off human controls	positive 2019-nCoV IgM, negative IgM		positive 2019-nCoV IgG, negative IgG			inactivated, cell-free, human blood-derived material, reactive for anti-SARS-CoV-2 IgG	serum pool
**Assay**	**Biozek COVID-19 IgG/IgM Rapid cassette**		**ENCODE COVID-19 IgG/IgM Rapid test device**		**Lumiquick rapid test**		**Euroimmun IgA/IgG_Anti-SARS-CoV-2**				**Vircell IgM+IgA/IgG**		**MAGLUMI 2019-nCoV IgG/IgM**					**Abbott SARS-CoV-2 IgG**	**Elecsys Anti-SARS-CoV-2**
Cross- reactivity evaluation	not-specified		not-specified		not-specified		SARS-CoV(-1), MERS-CoV, HCoV-229E, HCoV-NL63, HCoV-HKU1, or HCoV-OC43.			SARS-CoV(-1), MERS-CoV, HCoV-229E, HCoV-NL63, HCoV-HKU1, or HCoV-OC43.	Influenza virus A and B, adenovirus, *M. pneumoniae*, *Chlamydophila pneumoniae*, *Coxiella burnetii*, *Legionella pneumophila*, RSV	Parainfluenza virus 1, 2 and 3, influenza virus A and B, Aadenovirus, *M. pneumoniae*, *Chlamydophila pneumoniae*, *Coxiella burnetii*, *Legionella pneumophila*, RSV	Influenza virus A and B, parainfluenza virus, RSV, adenovirus, EBV, measles virus, CMV, VZV, *M. pneumoniae*, *Chlamydia pneumoniae, Monilia albicans*		Influenza virus A and B, parainfluenza virus, RSV, adenovirus, EBV, measles virus, CMV, VZV, M. pneumoniae, *Chlamydia pneumoniae, Monilia albicans*			*Borrelia burgdorferi, Candida albicans*, *Chlamydia trachomatis, Chlamydia pneumoniae*, CMV, *E.coli,* EBV, HAV, HBV, HCV, HEV, HIV, HSV, HTLV; Influenza, Listeria, measles, mumps virus, *Neisseria gonorrhoeae*, *Parvovirus* B19; *Plasmodium Falciparum,* Rubella, VZV *Treponema pallidum Toxoplasma gondii*	CMV, EBV, HAV, HBV, HCV, HDV, HSV, HTLV Type 1 and 2, RSV, Rubella, VZV, adenovirus, HIV, Influenza A and B, Influenza (unknown), *Picornavirus,* *E. coli, Toxoplasma gondii*
Inter- -ference	not-specified		not-specified		RF > 80 IU/ml, haemoglobin > 10 g/L; triglycerides > 37.0 mmol/L; bilirubin > 342 μmol/L		haemoglobin > 10 g/L; triglycerides > 22.6 mmol/L; bilirubin > 684 μmol/L				ANA interference, haemoglobin > 8.5 g/L; triglycerides > 22.6 mmol/L; bilirubin > 1026 μmol/L, cholesterol > 15 mmol/L, tributirin > 11 g/L, gamma globulins > 60 g/L, albumin > 60 g/L		HAMA > 30 ng/mL, RF > 1500 U/mL, bilirubin > 684 μmol/L triglycerides > 11.3 mmol/L, haemoglobin > 20 g/L, anti-mitochondria antibody > 1: 64 (titre), total IgG > 16.0 g/L, total IgM > 2.8 g/L, drugs					RF, HAMA, heterophilic antibodies	biotin > 1200 ng/mL, RF, haemolysis, bilirubin, pharmaceutical compounds
Sensitivity (depending of days after onset of symptoms, if available)	85%	100%	1-3 d: 16% 4-7 d: 87% > 8 d: 90%	1-3 d: 16% 4-7 d: 92.8% > 8 d: 90%	86.5%	85.1%	≤ 10 d: 44.8% 10-20 d: 100% ≥ 21 d: 100%		≤ 10 d: 22.4% 10-20 d: 87.5% ≥ 21 d: 100%		not specified		78.65%	91.21%			< 3 d: 0% 3-7 d: 50% 8-13 d: 91.18% ≥ 14 d: 100%		0-6 d: 65.5% 7-13 d: 88.1% > 14 d: 100%
Specificity	96%	98%	100%	100%	99.7%	99.3%	90.5%		99.3%		not specified		97.5%	97.33%			prepandemic samples: 99.6%; other respiratory illness: 100%		99.81%
Repro- -ducibility or within lab precision, CV %	not specified		not specified		not specified		depending on ratio: 6.5 to 16.1			depending on ratio: 5.7 to 16.2	depending on ratio: 3.93 to 4.74	depending on ratio: 3.92 to 4.95	neg. control: not reported; pos. control: 8.65%		neg. control: not reported; pos. control: 8.68%			neg. control: 5.9%; pos. control: 1.2%	not reported
Ag – antigen. ANA – antinuclear antibodies. CV – coefficient of variation. CLIA – chemiluminescence immunoassay. CMIA – chemiluminescent microparticle immunoassay. ECLIA – electrochemiluminescent immunoassay. CMV – Cytomegalovirus. EBV – Epstein-Barr virus. ELISA – enzyme-linked immunosorbent assay. HAMA – Human anti-mouse antibody. HAV – Hepatitis A virus. HBV – Hepatitis B virus. HCV – Hepatitis C virus. HDV – Hepatitis D virus. HEV – Hepatitis E virus. HIV – Human Immunodeficiency virus. HSV – Herpes Simplex virus. HTLV – Human T-Lymphotropic virus. LFIC – lateral flow immunochromatographic assays. NA – non-applicable. RF – rheumatoid factor. RSV – Respiratory syncytial virus. VZV – Varicella Zoster virus. d – days.																			

Eight borderline positive results obtained with Vircell IgM+IgA/IgG ELISA assay were excluded from statistical analysis since we were not able to repeat the test according to the manufacturer’s suggestion. Lateral flow immunochromatographic tests were performed by 4 operators and the result was always interpreted by the same operator who performed the test. In the case of a faint band, the result was defined by the consensus between two observers to minimize the visual error effect.

#### Real-time reverse transcription PCR

Ribonucleic acid was extracted from clinical specimens (nasopharyngeal swabs) with the viral RNA mini kit (Qiagen, Hilden, Germany) following the original manufacturer protocol.

The presence/absence of E and RdRp genes was determined by RT-PCR using the protocol previously published by Corman *et al.* (*15*). Briefly, a 20 µL reaction contained 5 µL RNA and 15 µL reaction mix (5 µL 4x TaqMan fast Virus 1-Step Master Mix (Applied Biosystems, Vilnius, Lithuania); E_Sarbeco_F1 and E_Sarbeco_R2 (final concentration 400 nM); E_Sarbeco_P1 (final concentration 200 nM); RdRP_SARSr_F2 and RdRP_SARSr_R1 (final concentration 400 nM); RdRP_SARSr_P1 and RdRP_SARSr_P2 (final concentration 200 nM)). All primers were synthesized by Invitrogen (Darmstadt, Germany) while probes were provided by AB (Applied Biosystems, Vilnius, Lithuania). Real-time reverse transcription PCR was performed on ABI 7500 (Applied Biosystems, Vilnius, Lithuania) using the following protocol: reverse transcription 5 min at 50 °C followed by 20 s at 95 °C and then 45 cycles of 15 s at 95 °C and 30 s at 58 °C. All patient samples, negative and positive controls were done in two replicates for both tested genes.

### Statistical analysis

For all tests, specificity and sensitivity with corresponding 95% confidence intervals (95%CI) were calculated. Kappa statistic was calculated to investigate agreement in positive/negative categorization between tests. Interpretation of Cohen’s kappa coefficient is as follows: 0.0–0.20 no agreement; 0.21–0.39 minimal agreement; 0.40–0.59 weak agreement; 0.60–0.79 moderate agreement; 0.80–0.90 strong agreement; > 0.90 almost perfect agreement (*16*). As ELISAs and chemiluminescence test results could be reported as a numerical value, we performed receiver operating characteristic (ROC) curve analysis to investigate whether acceptance of the cut-offs obtained by the ROC curve analyses could improve the diagnostic performance of the assays in comparison to manufacturer provided cut-off (*17*). Statistical analysis was performed on the whole group of RT-PCR positive individuals and those with > 10 days of symptoms duration but not on those with < 10 days of symptoms duration due to the small number of cases (N = 18). P value less than 0.05 was considered statistically significant. Statistical analysis was performed using the Medcalc Statistical Software version 19.1.5 (MedCalc Software Ltd, Ostend, Belgium).

## Results

Overall sensitivity for IgM ranged from as low as 28% up to 80%, for IgG from 76% up to 91%, and for IgA 87%. When the duration of symptoms > 10 days was taken into consideration, improvement of sensitivity was obtained for all assays. Specificity for IgG ranged from 85% to 100% and was generally higher in comparison to other isotypes from the same manufacturer, while for IgM ranged from 90% to 100%, and for IgA, it was 90%. Sensitivities and specificities for each assay, including the combination of isotypes, are presented in Table 2.

**Table 2:** Sensitivities and specificities of eight SARS-CoV-2 serological assays, including combined immunoglobulin isotype detection

**Method**	**Assay**	**Immunoglobulin isotype**	**Sensitivity (%)** **(95%CI)**	**Sensitivity (%)** **(95%CI)** **> 10 days of symptoms duration**	**Specificity (%)** **(95%CI)**
LFIC	ENCODE COVID-19 IgG/IgM Rapid test device	M	80 (66–91)	89 (71–98)	97 (83–100)
		G	80 (66–91)	89 (71–98)	100 (88–100)
		G + M	80 (66–91)	89 (71–98)	97 (83–100)
	Biozek COVID-19 IgG/IgM Rapid casette	M	28 (16–44)	30 (14–50)	90 (74–98)
		G	76 (61–87)	89 (71–98)	100 (88–100)
		G + M	76 (61–87)	90 (71–98)	90 (74–98)
	Lumiquick rapid test	M	47 (29–65)*	53 (29–76)^†^	90 (74–98)
		G	91 (75–98)*	100 (82–100)^†^	93 (78–99)
		G + M	91 (75–98)*	100 (82–100)^†^	90 (74–98)
ELISA	Euroimmun IgA/IgG Anti-SARS-CoV-2	A	87 (74–95)	96 (81–100)	90 (74–98)
		G	80 (66–91)	100 (87–100)	93 (78–99)
		A + G	94 (82–99)	100 (87–100)	83 (65–94)
	Vircell COVID‐19 ELISA IgM+IgA/IgG	A/M	96 (85–100)	100 (87–100)	70 (50–86)^‡^
		G	89 (76–96)^§^	96 (81–100)	85 (65–96)^║^
		A/M + G	96 (85–100)	100 (87–100)	64 (43–82)^¶^
CIA	MAGLUMI 2019-nCoV IgG/IgM	M	44 (29–59)	56 (35–75)	100 (88–100)
		G	87 (74–95)	93 (76–99)	100 (88–100)
		M + G	87 (74–95)	93 (76–99)	100 (88–100)
CMIA	Abbott SARS-CoV-2 IgG	G	78 (64–89)	96 (81–100)	100 (88–100)
ECLIA	Elecsys Anti-SARS-CoV-2	A/M/G	89 (76–96)	96 (81–100)	100 (88–100)
LFIC – lateral flow imunochromatography. ELISA – enzyme-linked immunosorbent assay. CIA – chemiluminescent immunoassay. CMIA – chemiluminescent microparticle immunoassay. ECLIA – electrochemiluminescent immunoassay. RT-PCR – reverse transcriptase polymerase chain reaction. *PCR-positive N = 32. ^†^PCR-positive > 10 days of symptoms duration N = 19. ^‡^PCR-negative N = 27 due to exclusion of the bordeline positives. ^§^PCR-positive N = 45 due to exclusion of the bordeline positives. ^║^PCR-negative N = 26 due to exclusion of the bordeline positives. ^¶^PCR-negative N = 25 due to exclusion of the bordeline positives.					

When the combined measurement of IgG, IgM, and IgA was taken into consideration in comparison to individual measurement, the improvement of sensitivities occurred for both ELISA assays but at the expense of specificity compared to the determination of IgG alone (Table 2). For CLIA assay, the diagnostic accuracy of combined measurement was equal to IgG alone, while for all LFIC assays did not improve the sensitivity and lowered the specificity in comparison to IgG alone. Agreement in positive/negative result categorization between assays is generally poorer for IgM (Table 3) than for IgG (Table 4) with the minimum level of agreement in 3, weak in 2, and moderate in only 1 case of comparison. Kappa coefficients for IgG agreement ranged from a moderate level of agreement in 10 comparisons, strong in 9, and almost perfect in 2 comparisons.

**Table 3:** Agreement of result categorization between assays for IgM isotype

	**Biozek COVID-19 IgG/IgM Rapid casette**	**Lumiquick rapid test**	**MAGLUMI 2019-nCoV IgG/IgM / Snibe**
	**Agreement (%),** **Kappa coefficient (95%CI)**	**Agreement (%),** **Kappa coefficient (95%CI)**	**Agreement (%),** **Kappa coefficient (95%CI)**
**ENCODE COVID-19 IgG/IgM Rapid test device**	63, 0.26 (0.09 to 0.44)	74, 0.46 (0.25 to 0.68)	76, 0.53 (0.36 to 0.70)
**Biozek COVID-19 IgG/IgM Rapid casette**	/	85, 0.63 (0.41 to 0.85)	74, 0.28 (0.03 to 0.52)
**Lumiquick rapid test**	/	/	69, 0.24 (-0.02 to 0.50)

**Table 4:** Agreement of result categorization between assays for IgG isotype

	**Biozek COVID-19 IgG/IgM Rapid cassette**	**Lumiquick rapid test**	**MAGLUMI 2019-nCoV IgG/IgM Snibe**	**Euroimmun IgA/IgG Anti-SARS-CoV-2**	**Vircell COVID‐19 ELISA IgM+IgA/IgG**	**Abbott SARS-CoV-2 IgG**
**Agreement (%),** **Kappa coefficient (95%CI)**	**Agreement (%),** **Kappa coefficient (95%CI)**	**Agreement (%),** **Kappa coefficient (95%CI)**	**Agreement (%),** **Kappa coefficient (95%CI)**	**Agreement (%),** **Kappa coefficient (95%CI)**	**Agreement (%),** **Kappa coefficient (95%CI)**
**ENCODE COVID-19 IgG/IgM Rapid test device**	90, 0.79 (0.65 to 0.93)	90, 0.81 (0.66 to 0.95)	96, 0.92 (0.83 to 1.00)	90, 0.79 (0.65 to 0.93)	90, 0.80 (0.66 to 0.94)	93, 0.87 (0.76 to 0.98)
**Biozek COVID-19 IgG/IgM Rapid cassette**	/	92, 0.84 (0.71 to 0.97)	91, 0.82 (0.69 to 0.95)	90, 0.79 (0.65 to 0.93)	86, 0.72 (0.57 to 0.88)	91, 0.82 (0.69 to 0.95)
**Lumiquick rapid test**	/	/	95, 0.90 (0.80 to 1.00)	89, 0.77 (0.62 to 0.93)	88, 0.75 (0.58 to 0.92)	89, 0.77 (0.62 to 0.93)
**MAGLUMI 2019-nCoV IgG/IgM Snibe**	/	/	/	88, 0.76 (0.62 to 0.91)	90, 0.80 (0.66 to 0.94)	92, 0.84 (0.72 to 0.96)
**Euroimmun IgA/IgG Anti-SARS-CoV-2**	/	/	/	/	87, 0.74 (0.58 to 0.90)	93, 0.87 (0.76 to 0.98)
**Vircell COVID‐19 ELISA IgM+IgA/IgG**	/	/	/	/	/	89, 0.77 (0.63 to 0.92)

ROC curve analysis revealed excellent diagnostic accuracy for IgG isotype for all four tested methods, with the area under the curve (AUC) ≥ 0.90 for all methods (Table 5). Also, the pairwise comparison of ROC curves did not show a significant difference between AUC values (P values from 0.086 to 0.894). When only the patients with symptoms duration > 10 days were included, the diagnostic accuracy of all four methods measuring SARS CoV-2 IgG isotype further improved, still without significant differences between methods (P values from 0.360 to 0.800).

**Table 5:** Receiver operating characteristic (ROC) analysis for methods measuring SARS CoV-2 IgG, IgA and IgM isotype, combination of IgA and IgM isotypes, and total antibodies

**Method**	**Symptom duration (days)**	**AUC (95%CI)**	**Sensitivity %** **(95%CI)**	**Specificity %** **(95%CI)**	**Optimal cut-off**	**Manufacturer** **cut off (units)**
**SARS CoV-2 IgG isotype**						
Abbott SARS-CoV-2	1–30*	0.96 (0.89–0.99)	85 (71–94)	97 (83–100)	> 0.4	≥ 1.4 (R)
	> 10	0.99 (0.93–1.00)	96 (81–100)	100 (88–100)	> 0.7	
Euroimmun Anti-SARS-CoV-2	1–30	0.90 (0.81–0.96)	78 (64–89)	100 (88–100)	> 1.3	≥ 1.1 (R)
	> 10	1.00 (0.93–1.00)	96 (81–100)	100 (88–100)	> 1.3	
Vircell COVID-19	1–30	0.93 (0.85–0.98)	84 (71–94)	100 (87–100)	> 11.5	> 6.0 (AI)
	> 10	0.99 (0.91–1.00)	96 (81–100)	100 (87–100)	> 11.5	
MAGLUMI 2019-nCoV	1–30	0.96 (0.89–0.99)	87 (74–95)	100 (88–100)	> 0.6	≥ 1.0 (AU/mL)
	> 10	1.00 (0.93–1.00)	100 (87–100)	93 (78–99)	> 0.4	
**SARS CoV-2 IgA, IgM and combination IgA and IgM isotypes**						
Euroimmun IgA Anti-SARS-CoV-2	1–30*	0.94 (0.86–0.98)	80 (66–91)	97 (83–100)	> 1.6	≥ 1.1 (R)
	> 10	0.97 (0.89–1.00)	93 (76–99)	97 (83–100)	> 1.6	
MAGLUMI 2019-nCoV IgM	1–30	0.76 (0.65–0.85)	59 (43–73)	100 (88–100)	> 0.6	≥ 1.0 (AU/mL)
	> 10	0.85 (0.73–0.93)	74 (54–89)	100 (88–100)	> 0.6	
Vircell COVID-19 IgM + IgA	1–30	0.94 (0.86–0.98)	80 (66–91)	100 (87–100)	> 14.1	> 8.0 (AI)
	> 10	0.99 (0.92–1.00)	100 (87–100)	89 (71–98)	> 10.3	
**SARS CoV-2 total antibodies**						
Roche Elecsys Anti-SARS-CoV-2 total antibodies	1–30	0.95 (0.88–0.99)	94 (82–99)	97 (83–100)	> 0.2	≥ 1.0 (COI)
	> 10	1.00 (0.94–1.00)	100 (87–100)	97 (83–100)		
*duration of symptoms in our PCR-positive group was 3–30 days but the group includes also asymptomatic individuals 1 day after the RT-PCR confirmation. AUC – area under the curve. R – ratio. AI – Antibody index. AU – arbitrary units. COI – cut-off index.						

ROC analysis for the methods measuring individual or combined markers of acute phase infection (IgA and IgM) revealed excellent diagnostic accuracy for IgA alone and a combination of IgA + IgM, while for IgM alone it was at the level of good accuracy (Table 5). In line with this, pairwise comparison of ROC curves showed significantly better diagnostic accuracy for IgA in comparison to IgM (P < 0.001) while combined measurement of IgA and IgM had no added value in comparison to IgA alone (P = 0.912). Diagnostic accuracy improved when only the patients with symptoms duration > 10 days were considered (Table 5). This especially refers to IgM, yet still significantly lower than IgA (P = 0.029).

To check if there is any added value of combined measurement of the markers of the acute and late phase of infection we included the Elecsys Anti-SARS-CoV-2 method in the pairwise comparison of ROC curves for IgG methods and did not observe any significant difference in the AUCs both for the whole RT-PCR-positive group (P values from 0.140 to 0.605) as well as for the group with > 10 days of symptoms duration (P values from 0.345 to 0.756).

Application of ROC analysis provided cut-offs improved diagnostic accuracy for assays: a) MAGLUMI 2019-nCoV IgM enabled detection of 7 more RT-PCR-positive patients irrespective of symptoms duration and 5 in the group with > 10 days of symptoms duration, without diminishing the specificity of the test; b) Euroimmun Anti-SARS-CoV-2 ELISA IgG change in cut-off nullifies the number of false positives to the detriment of the one false-negative case, irrespective of symptoms duration; c) Vircell COVID-19 IgG nullifies false positives without the change of sensitivity in the group with > 10 days of symptoms duration.

## Discussion

We aimed to compare diagnostic efficacy for detection of SARS-CoV-2 antibodies of eight commercially available serological assays of different formats. Our results confirmed the general poor utility of serological tests if performed in a period less than 10 days from the onset of the symptoms. The high variability between results of IgM assays was obtained, while the results for IgG showed good agreement and high diagnostic accuracy, especially after 10 days of symptoms onset. Low sensitivity of IgM isotype for one of the tested rapid devices pointed out the necessity for verification of the test before the use in patient care.

Implementation of SARS-CoV-2 IgM and/or IgA detection as a marker of an early immune response has been suggested as an additional diagnostic tool to RT-PCR test in patients with symptoms highly suggestive of COVID-19 infection but with negative RT-PCR test (*18*, *19*). To warrant this role, such a marker has to have a high sensitivity in the early disease phase in order not to miss potentially infective individuals. On our RT-PCR-positive group, IgM showed generally lower sensitivity in comparison to IgG of the same manufacturer, irrespective of symptoms duration. The exception is ENCODE COVID-19 Rapid test device for which IgM had the same sensitivity as IgG, and it was higher in comparison to other IgM assays. For this particular assay, sensitivities for both IgM and IgG obtained in our study group were very similar to those reported from the recent study (*20*). Similarly, sensitivities for IgA and IgG Euroimmun ELISA and IgM and IgG Maglumi CLIA in patients with > 10 days of symptoms duration are close to the sensitivities reported by other authors (*6*, *10*, *11*). In comparison to other assays, sensitivity for IgM Biozek COVID-19 LFIC assay was very low irrespective of symptoms duration. Similarly, Rudolf *et al.* also obtained a very low sensitivity of 19% (95%CI 12–28) for Biozek IgM for the post-symptom period of 7–28 days (*21*). The sensitivity of a serological test is influenced by several factors including antigen origin, antigen coating density, and serum dilution as well as observer visual error effect in the case of LFIC methods (*22*). The manufacturers did not specify antigen origin for either of the tested LFIC assays but sensitivity for IgG Biozek COVID-19 LFIC assay was comparable to all other tests so it is unlikely that the antigen origin could be the reason for exceptionally low sensitivity of IgM isotype. Also, the observer error effect was set to a minimum. Therefore, we speculate that employed serum dilution with sample buffer in relation to antigen coating density in Biozek COVID-19 LFIC assay may not be optimal for the detection of antibodies of IgM isotype.

Within our RT-PCR-positive group, there was no case with isolated IgM positivity detected with any of the tested assays. This observation, although noticed on the small study population, together with lower specificity of IgM obtained in comparison to IgG in LFIC assays, which is in line with the recent report highlighted the limited utility of IgM isotype detection in the acute SARS-CoV-2 infection (*23*). On the contrary, IgA showed better sensitivity in comparison to IgG when the whole RT-PCR-positive group was taken into consideration owing to the higher rate of positivity in the subgroup with < 10 days of symptoms duration. Within this subgroup, isolated positivity of IgA was detected in 6/18 (0.40) patients with the duration of the symptoms ranging from 4 to 9 days and in 2 asymptomatic patients tested 1 day after the RT-PCR-test. Additionally, the sensitivity of IgA rose to 96% after 10 days of symptoms, which confirmed the result from the recent study according to which IgA antibody can be reliably detected one week after the RT-PCR-confirmed SARS-CoV-2 infection (*24*). Higher diagnostic accuracy of IgA in comparison to IgM in our study was further confirmed with the lack of added value of combined detection of IgA and IgM in comparison to IgA alone in spite the fact that the assay which detects a combination of the isotypes employs whole inactivated antigen while the other assay employs S1 only. The use of serological markers in the acute phase of infection is overweighted with the high variability of the time of seroconversion as well as the magnitude of the immune response, which is associated with the disease severity (*22*). The high rate of false-negative results in the phase of the disease when patients are most infectious limits the role of serological analysis in diagnosing the acute infection (*25*). Testing for SARS-CoV-2 antibodies is more likely to be useful for determination of seroprevalence in population and identification of highly reactive potential human donors of convalescent plasma for therapeutic use (*26*, *27*). SARS-CoV-2 IgG antibodies which mostly appear after 10 to 14 days and lasts longer than IgM or IgA, fit this role. From the intended potential use, it is clear that such a serological test should have a high specificity with a minimal rate of false positives.

In this study, specificity < 95% was obtained for IgM Biozek LFIC assay (90%), Euroimmun IgA (90%) and IgG (93%) and especially low for Vircell (IgM + IgA 70% and IgG 85%). Manufacturer declaration regarding the crossreactivity study is given for both ELISA as well as for chemiluminescent assays but not for LFIC assays. For Euroimmun IgA and IgG assays, crossreactivity was examined for other coronaviruses, and pronounced crossreactivity was declared only with SARS-CoV-1. However, Charlton *et al.* obtained crossreactivity for Euroimmun assay with Parainfluenza virus (both IgA and IgG) and endemic coronaviruses 229E, NL63, and OC43 (IgA, only) (*14*). In the same study, no crossreactivity was observed for Abbott and Roche assays which are in line with 100% specificity for assays determined in our study. Crossreactivity examined by the manufacturer of the Vircell ELISA assay was performed on a small number of samples and did not include other coronaviruses, and yet confirmed some crossreactivity for IgM + IgA but none for IgG assay.

Diagnostic accuracy of the ELISA and chemiluminescence IgG assays did not significantly differ, including the Elecsys Anti-SARS-CoV-2 total antibodies assay which indicates the low added value of combined measurement of Ig isotypes in comparison to IgG, only. However, the number of RT-PCR-positive cases with symptoms duration less than 10 days included in our study is too small to assert this firmly.

It is worth noting that among 10 of our asymptomatic individuals sampled 1 day after RT-PCR confirmation, none was negative with all tests, and surprisingly, most of them were positive for both IgA/M and IgG with more than at least two tests of different formats.

Obtained sensitivities and specificities for five out of eight accompassed assays are similar to those declared by the manufacturers, taking into account the slight differences in the time from the onset of symptoms used for testing. Significant discrepancies were obtained for IgM isotype in two LFIC assays (Biozek and Lumiquick) and Maglumi CLIA assay. However, the time from symptoms onset used by these manufacturers for evaluation of sensitivities and specificities is unknown and can have a huge impact on comparability with our results.

Comparison of IgG serological assays in our study confirmed a low level of variability between different assays (including rapid tests), despite the differences in employed antigenic targets (S1 only, N only, or combination of both). On the contrary, variability between results of the IgM assays was high with only one acceptable agreement (between two rapid tests) according to the rule that kappa coefficient < 0.60 indicates inadequate agreement between methods to be equally employed in clinical laboratory practice (*16*). The low agreement between IgM assays could be attributed to the above-mentioned cross-reactivity of SARS-CoV-2 IgM with other viruses which is unknown data for the rapid tests included in the study. Also, the impact of the rheumatoid factor (RF) as the common cause of cross reactivity is mostly unknown for rapid tests.

Improvement of diagnostic performance with the acceptance of ROC analysis provided cut-offs for some assays pointed out the importance of the verification of manufacturer-provided cut-off values on the local population. The choice of the cut-off is highly dependent on the intended use of the assay with the imperative for a minimum of false-positives for detection of the acquired immunity (*27*). Also, the results of our study suggest that the choice of the cut-off might be related to the time from the symptoms onset or RT-PCR test for asymptomatic individuals.

Despite the prejudices of the unreliability of SARS-CoV-2 rapid serological tests, the results of our study showed that the reliability is manufacturer-dependent. LFIC assay format is easy to handle but the important drawback of this technology is the high impact of the test performer on the result, including the manipulation with the sample and buffer and, most important, the interpretation of the faint bands. Although we tried to minimize the bias in interpretation, significant discrepancies between manufacturer declared sensitivities and those obtained in our study for two LFIC assays could be partly attributed to this problem.

Advantages of rapid assays are easy handling, no need for extra instrumentation, low volume of sample, the possibility of the whole blood application, and fast results. On the other hand, advantages of conventional assays are automation, avoidance of the prolonged exposure to biological material, available quality control that assures batch-to-batch reproducibility and results that are not prone to subjective interpretation. Numeric results could provide added value in the assessment of the intensity of the immunologic response and also allow the adjustment of the cut-off according to ROC analysis performed on the representative population.

The strengths of this study are related to the experimental design and effort to compare eight differently designed assays (rapid and conventional, manually and fully automatic). The majority of studies presented sensitivities and specificities of different SARS-CoV-2 serological assays while our study additionally evaluates the agreement of the results between tested assays.

Although this is not the first study that compares several different serological SARS-CoV-2 assay methods, to the best of our knowledge, Lumiquick IgG/IgM and Biozek IgG/IgM as rapid assays and Vircell IgM+IgA/IgG as an ELISA method were not previously compared to other assays included in this study.

The low number of participants, especially those with a duration of the symptoms less than 10 days is the limitation of the study. Additionally, to determine the specificity of the SARS-CoV-2 serological assay, the use of prepandemic samples would be the most correct approach.

In conclusion, our study confirmed that the selection of the appropriate time-frame for testing is crucial for the proper investigation of immunity, which can be very challenging among asymptomatic persons. There is high variability between IgM SARS CoV-2 serological assays independently of the assay format. On the contrary, IgG assays showed moderate to perfect agreement. We observed a higher diagnostic accuracy of IgA in comparison to IgM SARS CoV-2 serological assays.
